# Effect of Propylene Glycol on the Sealing Ability of Mineral Trioxide Aggregate and Calcium-Enriched Mixture Cement Apical Barriers

**DOI:** 10.22037/iej.v12i3.15670

**Published:** 2017

**Authors:** Alireza Adl, Fereshte Sobhnamayan, Nooshin Sadat Shojaee, Fateme Tahmasebi Azad, Mohsen Bahmani

**Affiliations:** a *Department of Endodontics, Biomaterials Research Center, Dental School, Shiraz University of Medical Sciences, Shiraz, Iran; *; b *Department of Endodontics, Dental School, Shiraz University of Medical Sciences, Shiraz, Iran; *; c *Dental School, Shiraz University of Medical Sciences, Shiraz, Iran*

**Keywords:** Apical Plug, Calcium-Enriched Mixture Cement, Mineral Trioxide Aggregate, Propylene Glycol, Sealing Ability

## Abstract

**Introduction:**

Propylene glycol (PG) improves the handling, physical, and chemical properties of mineral trioxide aggregate (MTA). This study aimed to evaluate the effect of PG on the sealing ability of MTA and calcium-enriched mixture (CEM) apical barriers.

**Methods and Materials:**

A total of 70 extracted human maxillary single-rooted teeth were prepared using ProTaper rotary system. The apical 3 mm of the root tips were resected and the root canals were enlarged with Peeso reamers up to #4, to create open apex teeth. The teeth were then randomly divided into four experimental (*n*=15) and two control (*n*=5) groups. Group1: MTA+ MTA liquid, group2; MTA+MTA liquid (80%) + PG (20%), group3; CEM+CEM liquid, group4; CEM+ liquid (80%) + PG (20%). Cements were mixed with their respective mixing agents and a 4-mm thick apical plug was fabricated. The microleakage was measured on day 1, 3, 7 and 21 using a fluid filtration technique. The repeated measures ANOVA and Sidak test were used to analyze the data.

**Results:**

All experimental groups demonstrated various amounts of microleakage. No significant difference was found between MTA and CEM cement (*P*=0.193), regardless of time and liquid components. There was no significant difference was observed between liquids (*P*=0.312) in all time intervals. The rate of microleakage decreased over time and a significant differences was observed between all intervals (*P*<0.05), except 3-7 and 7-21 (*P*=0.190) days.

**Conclusion:**

PG demonstrated neither a positive nor a negative effect on the sealing ability of Angelus MTA and CEM cement.

## Introduction

Endodontic management of a non-vital immature tooth is complicated, due to extremely wide apical foramen and there is no barrier against which obturation material can be compacted [1-3]. Long-term calcium hydroxide has been traditionally used to establish apical closure by induction of a hard tissue barrier [4-6]. However, this method requires multiple appointments and radiographies, and is also associated with some difficulties, such as potential for tooth fracture or coronal leakage [6-8]. The artificial apical barrier technique has been proposed as an alternative to long-term calcium hydroxide apexification [6]. This technique has been described as creating an artificial apical stop by non-surgical compaction of a biocompatible material into the apical end of the root canal; thus enabling filling the root canal [9].

Application of mineral trioxide aggregate (MTA) as an artificial apical barrier has a rather long history [10]. Many studies have reported the clinical success of artificial root-end closure using MTA [8, 11-13]. The popularity of MTA as an artificial apical barrier has been attributed to its biocompatibility, ability to induce hard tissue formation and good sealing properties [8]. Despite these advantages, MTA has a long setting time, tooth discoloration potential and poor handling properties [14]. Moreover, controversy exists with respect to its ability to provide apical seals in apexification procedures [15]. It has been suggested that mixing MTA with propylene glycol (PG) may improve the handling, physical, and chemical properties of MTA [16-19]. PG is a viscous alcoholic compound approved by the Food and Drug Administration as a safe food additive. Holland *et al.* [17] showed that PG facilitates the placement of MTA into root canals without influencing its biocompatibility. It has also been shown that the use of PG as a vehicle for gray Angelus MTA increased its sealing ability in furcal perforations [18]. Furthermore, mixing MTA with PG increases its push-out strength to dentin [19].

Calcium-enriched mixture (CEM) cement is another hydrophilic cement successfully used as an artificial apical barrier in open apex teeth [20]. Like MTA, CEM cement is biocompatible and has favorable sealing ability [21]. As a root-end filling material, CEM cement is capable of inducing periradicular tissue healing regeneration, including the production of cementum and new bone [22]. Considering the promising effect of PG on the sealing ability of MTA in furcal perforation [18], this study was designed to evaluate the effect of PG on the sealing ability of CEM cement in comparison with MTA as artificial apical barriers in open apex teeth.

## Materials and Methods

A total of 70 extracted human maxillary single-rooted teeth were collected. After extraction, the teeth were placed in 1% sodium hypochlorite for 48 h, to be disinfected, and were then rinsed and stored in normal saline solution until used. The teeth were examined for cracks and calcified canals. The selected teeth were decoronated, so as to have a standardized length of 12 mm. 

The root canals were cleaned and shaped using ProTaper rotary system (Dentsply Maillefer; Ballaigues, Switzerland) up to F3 according to the manufacturer’s protocol. During instrumentation, after each rotary file, 2 mL of 2.5% NaOCl was used. The apical 3 mm of the root tips were then resected perpendicular to the long axis of teeth. To create open apex teeth, the canals were instrumented with #4 Peeso reamers (Mani, Tochigi, Japan) with each instrument passing 1 mm beyond the apex [23]. To remove the smear layer, canals were filled with 3 mL of 17% ethylenediaminetetraacetic acid (EDTA) (Ariadent, Tehran, Iran) and 1 mL of 2.5% NaOCl for 3 min, each. Final irrigation was performed with 5 mL of normal saline. According to the cement types and mixing agents, the teeth were randomly divided into four experimental (*n*=15) and two control (*n*=5) groups: group 1; MTA+MTA liquid, group 2; MTA+ MTA liquid (80%)+ PG (20%), group 3; CEM+CEM liquid and group 4; CEM+ liquid (80%)+ PG (20%).

White MTA (Angelus; Londrina, Parana, Brazil) and CEM cement (BioniqueDent; Tehran, Iran) were prepared according to the manufacturers’ instructions. The instrumented canals were dried with paper points (Gapadent, Xinkou, China) and the samples were fixed in flower mounting sponges. Moist cotton was placed opposite the apices to simulate periapical soft tissues. MTA and CEM cement were carried into the canals with MTA carrier (Dentsply Maillefer, Ballaigues, Switzerland) and condensed with a hand plugger to achieve a 4-mm-thick apical plug. Thickness of the apical barrier was confirmed by radiographs. Then, moistened paper points were placed in the canals and samples were stored in 37^º^C and 100% humidity for 24 h.

In the negative control group, the canals were filled with MTA and their apical ends were sealed with sticky wax. In the positive control group, the prepared canals were left unfilled. In the experimental and positive control groups, the entire root surface was coated with two layers of nail varnish, except the area corresponding to the resected root-end surface. In the negative control group, the entire root surface, including the apical opening, were covered with two layers of nail varnish.

Microleakage was evaluated using the fluid filtration technique, employing a pressure of 20 cm H_2_O [24]. In this method, a device was designed by attaching two micropipettes perpendicular to each other, using a plastic connector. A plastic tube was also used to connect the apical part of the root to the end of the horizontal micropipettes with cyanoacrylate glue (Razi, Tehran, Iran). All connections of the testing apparatus were also tightly closed with the cyanoacrylate glue.

**Figure 1:**
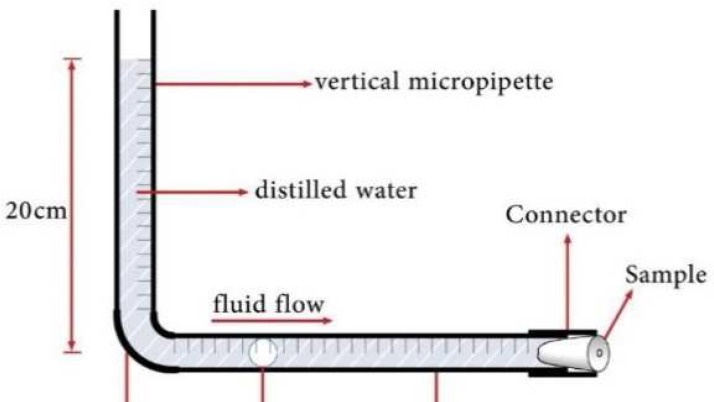
A schematic view of design fluid filtration system

The pipettes and the plastic tubes at the apical sides of the specimens were filled with distilled water. To apply a pressure of 20 cm H_2_O, the vertical micropipette was filled just to 20 cm. A small air bubble was introduced into the system with a syringe and advanced into the horizontal micropipette (Figure 1)

The volume of the fluid transport was measured by observing the movement of the air bubble. Microleakage for each sample was measured at day 1, 3, 7, and 21. The rate of microleakage (RML) of each interval (0-1, 1-3, 3-7, 7-21 days) was calculated and expressed in μL/min [25] (bubble displacement in each interval/period of interval).

***Statistical analysis***

The repeated measures ANOVA test was employed to analyze the change of sealing ability over time. For pairwise comparisons, the Sidak test was used*. P*-value was considered significant at the level of 0.05 for all tests.

## Results

While the positive control group showed the maximum amount of leakage; the negative control group showed no leakage (Table 1). All experimental groups demonstrated various amounts of microleakage. There were no interaction effects between materials and time (*P*=0.252), vehicle types and time (*P*=0.578), and materials and vehicle types (*P*=0.938). 

Regardless of time and vehicle type, no significant difference was found between MTA and CEM cement (*P*=0.193).

Regardless of time and material, there was no significant difference between vehicles (*P*=0.312). The RML decreased over time. There were significant differences between time intervals (*P*<0.05), except 3-7 and 7-21 days (*P*=0.190).

## Discussion

This study compared the microleakage of MTA and CEM cement as apical barriers when mixed with PG. Various methods have been used to evaluate the sealing ability of apical plugs, such as dye leakage, bacterial leakage, glucose penetration, radioisotope labeling, electrochemical method and fluid filtration technique [26].

The dye penetration method is widely used but has potential problems. Particle molecular size, pH, and chemical reactivity of the dyes may affect their degree of penetration [26]. Moreover, it has been shown that alkaline materials, such as MTA and CEM cement, have the potential to discolor the methylene blue dye, which may lead to unreliable findings [27].

Bacterial leakage methods are clinically and biologically more relevant than dye leakage methods [28]. However, these methods are qualitative in nature and one passing bacteria through the root canal can multiply and cause positive culture and turbidity [26]. In addition, if tested materials have antimicrobial properties, it is irrational to employ such methods [26].

Another method introduced for endodontic leakage studies is based on the filtration rate of the glucose along the filled root canals [29]. However this model is inappropriate for evaluating the sealing ability of alkaline materials because glucose in alkaline solutions is slowly oxidized; forming gluconic acid that finally converts to gluconate and glucose detection kits cannot detect gluconate [30].

The fluid filtration system used in the present study is a reliable technique to measure microleakage, and is widely used to test the sealing ability of different kinds of restorative and root canal filling materials [31-34]. This technique provides a quantitative measurement of microleakage over a time without destruction of experimental specimens [35].

To evaluate the effect of PG on the sealing ability of MTA and CEM cement, in this study, 20% PG was added to the associated liquid of each cement specimen. Duate *et al.* [4] in their study on some physical and chemical properties of MTA recommended this ratio because it had the least effect on the setting time of MTA while improving the flowability and increasing the pH and calcium ion release of the cement. Milani *et al.* [5] also showed that this ratio increased the push-out bond strength of MTA to dentin.

The results of the present study showed that PG has no effect on the sealing ability of MTA and CEM cement. This finding is inconsistent with that reported by Brito-Junior *et al.* [18], who showed that the use of PG as a vehicle for gray Angelus MTA increased its sealing ability in furcal perforations.

Direct comparison of the two studies cannot be performed because of the different experimental methodologies, including the leakage test (fluid filtration versus bacterial leakage), type of material (white MTA and CEM cement versus gray MTA), and different proportion of PG (20% versus 100%). Therefore, further research is required to determine the influence of different proportions of PG on the sealing ability of endodontic cements.

Although in this study PG had neither promising nor adverse effects on the sealing ability of MTA and CEM cement, other studies support mixing MTA with PG for improving its handling [17], bond strength to dentin [19], flowability, pH, and calcium ion release [16]. Therefore, the authors have no reason to reject mixing MTA with PG.

On the other hand, considering the fact that no document is available that shows PG has any positive impact on the other properties of CEM cement, the authors do not recommend mixing CEM cement with PG until further studies are undertaken. 

**Table 1.** Mean (SD) of microleakage (µL/min) in different groups

**Group **	**0-1 days**	**1-3 days**	**3-7 days**	**7-21 days**
**MTA**	0.78 (1.43)	0.3 (0.22)	0.15 (0.16)	0.12 (0.16)
**MTA+PG**	0.97 (1.3)	0.57 (0.35)	0.26 (0.26)	0.19 (0.13)
**CEM**	1.25 (1.8)	0.52 (0.54)	0.27 (0.23)	0.13 (0.06)
**CEM+PG**	1.6 (2.49)	0.76 (1.29)	0.24 (0.21)	0.16 (0.11)

In the current study, regardless of cement and vehicle types, the rate of microleakage decreased over time. This is consistent with the results of previous studies [5, 11, 12]. Gancedo-Caravia and Garcia-Barbero [36] showed that, under wet conditions, the push-out bond strength of MTA increased over time, up to 21 days. This is consistent with the results of the present study, in which improved bonding to dentin leads to better sealing.

Another finding of this study was that Angelus MTA and CEM, as apical barriers, have comparable sealing abilities. Similar results have been reported in previous studies [2, 8, 10, 13, 37-39]. The high sealing ability of MTA and CEM cement can be explained by the slight expansion of two cements during setting [40-42], resulting in enhanced adaptation of cement to the dentinal walls. In addition, MTA and CEM cement are able to form hydroxyapatite, which may improve the seal at the interface of cements and dentin walls [2, 43, 44].

## Conclusion

Under the limitation of this *in vitro* study, PG had no positive or negative effects on the sealing ability of Angelus MTA and CEM cement. Therefore, the recommendation of mixing with PG depends upon its effect on the other properties of MTA and CEM cement.
